# The Hospital. Nursing Section

**Published:** 1904-10-08

**Authors:** 


// The Hospital.

Nursing Section.

Contributions for this Section of "THE HOSPITAL" should be addressed to the EDITOR, "THE HOSPITAL," NURSING SECTION, 28 & 29 Southampton Street, Strand, London, W.C.

No. 941.—VOL. XXXVII. *SATURDAY, OCTOBER* 8, 1904.

### Notes on News from the Nursing World.

### THE JAPANESE GOVERNMENT AND AMERICAN NURSES.

WHEN the war between Japan and Russia broke out, we were in a position to authoritatively inform our readers that the Japanese Government did not desire the assistance of any foreign nurses, English or otherwise. We believe that so far as English nurses are concerned this assurance was generally accepted. In the United States, however, a different view of the matter prevailed, and a certain number of American nurses persisted in going to Japan. They were, of course, received with courtesy, and even with compliments, by the polite officials at Tokyo. But, as the *New York Medical Record* now frankly states, they were not wanted, and in the words of our contemporary, they "have been a source of the greatest annoyance and anxiety to the Government." Our contemporary goes on to say, "While grateful for the self-sacrifice shown in travelling so many thousand miles to nurse wounded and sick Japanese soldiers and sailors, the Medical Department of the Japanese Army has been frightfully embarrassed by their attentions," and declares that their ignorance of the Japanese language, their inability to eat Japanese food, or to live in Japanese style, has proved an almost complete bar to their usefulness and a source of great trouble. Last of all, it adds, "The nurses were not needed, as Japan possesses an army medical organisation unsurpassed, and probably unequalled, by that of any other nation." This is what we stated when the question of foreign nurses going to Japan first arose. This experience is a repetition of incidents in the Græco-Turkish War. Nurses should show more discipline when wars occur, or they may come to be classed with the "plague of women" type.

### PRINCESS CHRISTIAN'S VISIT TO CAPE TOWN.

IN the two days which Princess Christian spent in Cape Town she had very little time at her disposal to devote to nursing and hospitals. She arrived on Tuesday, September 6th, and left again on Friday, the 9th, but on the Wednesday morning her Royal Highness paid a purely informal visit to the Victoria Nurses' Institute, where, after being received by the members of the Committee, she made an inspection of the Institute. The nursing staff were then presented to her, and she expressed a wish to become a patroness of the Institute, and also consented to perform the ceremony of opening the new wing on her return from her visit to the Victoria Falls. On Thursday, despite the wet and stormy weather, Princess Christian went by motor-car to Wynberg, where she inspected the Military Hospital, and also the Victoria Cottage Hospital, going through all the wards, and saying a few kindly words to the inmates Before leaving Cape Town she sent a large panel photograph of herself to all the institutions she visited.

### THE "STAGE ARMY" AT WORK IN AUSTRALIA.

WE regret to learn that, in common with Miss L. L. Dock, secretary of the International Council of Nurses, the Royal Victoria Trained Nurses' Association, has been imposed upon by the "stage army," who affected to represent the nursing world in Great Britain at the Congress in Berlin. The organ of the Association, in publishing an account of the Congress, presumably supplied by the lady who, in the absence of any Victorian nurse, was asked to represent the Association, affirms that "the Berlin meeting was in every sense a representative one." It would be a pity if an assertion so misleading and ridiculous in respect to Great Britain, were accepted by nurses at the Antipodes; and we therefore repeat, for the benefit of our numerous readers in the colony of Victoria, the statement that the representative superintendents and working nurses of this country did not attend the Berlin Congress, and that, as a matter of fact, only a small clique advertising themselves under the names of imposing organisations, all consisting of the same persons, took part in the proceedings.

### THE FUNCTIONS OF THE CENTRAL MIDWIVES BOARD.

A POINT of considerable interest was raised at the first meeting of the Central Midwives Board after the holidays. The medical superintendent of one of the workhouse infirmaries asked the Board for advice as to the position of new lying-in wards. Dr. Sinclair, contending that such questions were outside the scope of the Board, desired that a reply to that effect should be forwarded. The other members, however, took the view that when the Board were requested to furnish advice they should give it if possible, and it was decided to recommend the correspondent to inspect the lying-in wards at Kensington Workhouse Infirmary. Dr. Sinclair also protested against any notice being taken by the Board of a communication from another official who urged an amendment of the Act for the purpose of giving power to local authorities to grant compensation to midwives suspended from practice under one of its sections. His contention is that the functions of the Board are simply administrative, and, perhaps, he may be technically correct. But if the other members take a broader view of their responsibilities and consider that it is not outside the scope of them to render reasonable assistance and not to ignore well-intended suggestions, we do not think that they will expose themselves to adverse comment.

### SHAMEFUL NEGLECT OF THE MENTALLY AFFLICTED IN IRELAND.

The report of inspectors of lunatics for 1903, which has just been presented to the Lord-Lieutenant of Ireland, shows that nothing less than a revolution in the system of nursing the mentally afflicted poor in the sister island is required. Of course, this could only happen as part of a sweeping scheme of reform in the administration of the Poor-law, but the urgent need of proper nursing is disclosed in two or three sentences of the report. At one workhouse the patients are described as wearing neither shoes nor stockings; at another, the hair of one was encrusted with tea-leaves and dry bread, as the result of her habit of pouring tea, with bread broken in it, on her head, from time to time; at a third, the bed-ridden patients were in a neglected state, and the sheets and quilts were dirty. This sort of thing would be impossible if skilled nurses, instead of ignorant attendants, were employed; and we trust that Lord Dudley, whose wife has done so much to benefit the sick poor in the sparsely populated districts, by providing them with trained nurses, will bring all the influence he can to bear upon the authorities of the Irish union workhouses in order to secure for the mentally afflicted the care and attention to which they are apparently strangers.

### A CURIOUS IDEA OF FULL UNIFORM.

The question of what constitutes full uniform is raised by a correspondent in a letter elsewhere, who cites the case of several nurses who joined an institution under an agreement to receive a certain salary, and "full indoor and outdoor uniform." When it came to actual experience each nurse found that the phrase only meant material for dresses and cloaks, and one bonnet. Neither caps, collars, cuffs, nor aprons were forthcoming; and, of course, the expense of making dresses and cloaks, together with trimming and extras, had to be defrayed by the newly-engaged nurse. It is impossible to justify the misleading description by the authorities of the institution in question, and it would have served them right if one of the nurses had proposed to attend her first case with the yards of material, given her as "uniform," arranged gracefully around her with the aid of a few pins. Such things, however, would not be possible if nurses would learn to be more businesslike. It is not merely their right, but their duty, to institute full inquiries respecting the conditions of any engagement upon which they propose to enter. If the nurses in this instance had asked for the details of the "full uniform" supplied, they would have avoided their present difficulty by either declining the appointment, or by stipulating for a salary sufficient to cover deficiencies.

### THE TEACHING OF MASSAGE.

On Monday the National Hospital for the Paralysed and Epileptic opened a school for the teaching of massage. The arrangements for the establishment of the school have been in the hands of a committee appointed by the medical staff and the board. It will be held in the hospital, and is under the direction of Madame Gripenwald. Only female pupils have entered for the present term, but it is proposed to appoint a male instructor and to take male pupils when a sufficient number have entered their names. The chairman of the hospital in addressing the pupils said that the National Hospital had for some years taught massage, but that the gradual adoption of the Swedish movements by the medical profession in England had now rendered it necessary to extend the teaching in order that the holders of National Hospital certificates might be recognised as competent to perform all such ordinary duties as might be required of them by English physicians and surgeons. The committee had appointed a competent Swedish lady to instruct the pupils and to present them for examination, and the object of the doctors who would examine them would be to make sure that they were competent to carry out the instructions which were now commonly given by English physicians to medical rubbers. A certain number of the nurses of the hospital will be trained in the school free of charge.

### SEA-GOING NURSES.

The movement for supplying nurses for liners, irrespective of the stewardesses, is still, Miss Penn assures us, proceeding. That is to say, she continues to receive letters from all parts of the world in favour of it, and she states that up to the present 140 trained nurses have offered their services since she set the ball rolling. But in the existing condition of the shipping trade, she adds, it is useless to ask shipowners to do anything. We are glad to learn that this does not discourage her, and that she intends to prosecute her efforts with renewed vigour when the auspices are more favourable. There can be no doubt as to the need of trained nurses on board ships bound for tropical voyages. In fact, as the serious illness of the wife of the Bishop of Ripon in crossing the Atlantic indicates, they would be serviceable on other vessels. It remains for pioneers like Miss Penn to keep pegging away, and we think that it would be good policy for the shipowners, even in bad times, to pay heed to her representations.

### THE NURSING OF CRIPPLED CHILDREN.

The Lady Mayoress of Manchester opened on Saturday a Country Nursing Home which has been erected for the benefit of crippled children at Cross Lane, Marple. The home is situated in a delightful part of the country, is under the control of a representative committee in Manchester, and is in charge of a trained hospital nurse. At present there is only accommodation and provision for nine inmates, but we do not doubt that when the advantages which the children derive from being under the care of a trained nurse are recognised, the Committee will find that Lancashire people are prepared to enable them to extend the scheme. As the author of an article which appeared in our columns a fortnight ago observed, "In the nursing of crippled children the sympathies of a tender-hearted woman are touched as they cannot be in any other department of nursing." We rejoice whenever these sympathies are afforded fresh opportunities of manifesting themselves.

### PROGRESS AT ALNWICK.

It is stated in the annual report of the Alnwick District Nursing Association, just issued, that during the 12 months the nurses attended 164 medical cases, 100 surgical cases, 17 maternity cases, and nine chronic cases, and that they paid 7,718 visits. The financial position is satisfactory, the balance in hand after the payment of all expenses being £114. The Duke of Northumberland subscribed £50, and the Duchess £28 10s. But even without these contributions the organisation would have been in a fairly flourishing condition, thanks to the increase in the number of small subscribers who benefit by the work of the nurses.

### BRAVERY OF A LIVERPOOL NURSE.

At an inquest in Liverpool on the body of a domestic servant, who was burnt to death, evidence was given showing that a nurse in the next house behaved with great courage and presence of mind. Hearing the cries of the unfortunate young woman she ran to her and with difficulty managed to extinguish the flames with her hands, burning several of her own fingers in the effort. The deceased was so badly injured that she died at the Royal Infirmary the next day, and the jury having returned their verdict the Deputy Coroner said that he could not allow the case to pass without referring to the brave conduct of the nurse. The jury concurred in his remarks.

### TRAINED NURSES AND MERIDEN INFIRMARY.

There is so frequently a difficulty in obtaining the services of trained nurses at workhouse infirmaries that we are glad to be able to mention a different kind of experience. The authorities of Meriden, in Warwickshire, advertised a vacancy, and received 28 applications from trained nurses. The infirmary is not a large one, and the number of answers from qualified persons suggests that nothing to the detriment of the institution is known in the nursing world.

### THE UNQUALIFIED MIDWIFE.

In spite of the operation of the Midwives Act the unqualified midwife is still abroad in the land. At Hammersmith last Friday an inquest was held into the death of a woman who had been attended by a person of this description. It transpired in the course of the evidence that though immediately after the confinement of the deceased bad symptoms were apparent the "midwife" did not send for a doctor for over an hour. When he arrived the patient was dying. The "midwife" candidly admitted that she did not know what to do under the circumstances, and we are not surprised that the jury found that the patient's death was accelerated by her neglect and ignorance. They considered that "she should be severely censured for her incompetency." It would have been as well to have added a warning that if she repeats her display of incompetence with like results, she will not escape so lightly.

### A FAREWELL RECEPTION.

On Tuesday, last week, in the board-room of the temporary premises of the Liverpool Children's Infirmary, a reception was held by the matron for the purpose of bidding farewell to Miss Emily Munroe. For the last 12 years "Sister Emily" has rendered valuable service to this institution, where her work will be more particularly remembered in relation to the out-patient department, at whose doors over 60,000 patients seek relief annually, and in which day after day she has for many years in her capacity of out-patient sister toiled with exemplary ardour and signal success. There were present at the reception members of the committee, the honorary physicians and surgeons, and members past and present of the nursing staff, who, in token of their regard and esteem for the departing sister, presented her with numerous gifts. The gift of the committee was a gold and platinum necklace, with a pendant of pearls and inscription; of the honorary medical staff, a clock, suitably inscribed and accompanied by an illuminated address; of the matron and past and present members of the nursing staff, a silver tea-service; individual friends connected with the infirmary presented her with a china tea-service (cups and saucers), silver teaspoons, a silver frame, a cheque, a picture, and a silver-mounted purse; while the domestic staff gave her a glass butter-dish. Miss Munroe, in going from Liverpool to a superior post in Manchester, leaves behind her many grateful friends, not least among whom will be the hundreds of little children who have been for so long the object of her untiring care.

### THE BATHING DIFFICULTY.

In a recent article in our columns, describing a nurse's work in an Irish hospital, the writer mentioned that one of the duties which she found the most difficult was to overcome her patients' dislike to soap and water. That this antipathy, though said to be especially strong in the Irish poor, is pronounced in some Englishwomen, was proved in rather a sad manner last week at the Islington Workhouse Infirmary. An old "Granny" of 96 was to have a bath and she resisted the operation with all the strength of which the poor dirt-loving body was capable. The struggle resulted in her arm being broken. Evidence given at the inquest following her death showed that, although it was primarily due to old age, her end had been accelerated by the shock to the system caused by the injury, for which the nurses were not, however, blamed.

### PAYMENT FOR TEMPORARY DUTY.

At a meeting of the Lewisham Guardians it was proposed that in future no temporary nurse should be employed at a higher wage than 15s. per week, without the sanction of the Board. The author of the proposition said that he submitted it because a temporary nurse had been paid 30s. Subsequently the matter was referred to the workhouse committee. We imagine that the committee will decide that the proposal is both impracticable and undesirable. In an emergency it might be impossible to obtain the sanction of the Board, and a trained nurse cannot be obtained for the sum of 15s. a week for temporary duty.

### SHORT ITEMS.

We are officially informed that Miss M. S. Ram, Miss M. F. Steele, and Miss E. Foster have been provisionally appointed staff nurses in Queen Alexandra's Imperial Military Nursing Service.—Miss A. S. Ravenshaw, formerly lady sanitary inspector to the borough of Bury, Lancashire, has accepted the position of matron of the private hospital, Garstang Road, Preston. Prior to her appointment at Bury, Miss Ravenshaw was for a few years ward sister at Crumpsall Infirmary.—Since the official announcement of the constitution of the nursing staff at the Northwood branch of the Mount Vernon Hospital, Hampstead, was made, it appears to have undergone revision. We are now informed that there is a night sister and six probationers.

### The Nursing Outlook.

"From magnanimity, all fear above;
From nobler recompense, above applause,
Which owes to man's short outlook all its charm."

### NURSING THE AGED.

There is a scheme before the Kingston Guardians to put all the aged and imbecile under the charge of the Infirmary Committee and the Infirmary staff, and to remove all the able-bodied to a farm and have a real "work"house there. It is a scheme which has humane and worthy points, for old people need nurses as much as children do, and we have always upheld that the mentally defective are peculiarly dependent on the help of the trained nurse who can realise their irresponsibility.

Even after all that has been said and written, and in spite of the presence of women guardians, the treatment of the old people in many of our workhouses is harsh and cruel, and some of the dear old grannies have wept bitter tears when sent back from the infirmary to the "house." For in the infirmary there was a nice bright young nurse who called her "Mother" and treated her with respect, and there was a kindly matron who came round with peppermints, and there was a sister—but ah! the story of that sister deserves telling more fully. For when Granny came to the Infirmary her age was given as 72, but Granny insisted she was really 77. "I was 72 when I came to the House, and they've kept me at that ever since, and I ain't never had a birthday, but it were five years ago I come, it were." And Granny wept. But Sister went away and made a plain cake with sugar on it, and put "77" on it, and brought it to Granny, and Granny had a birthday! All very childish perhaps, but then that is just the point, the very old are as childish as the very young, and they need sympathetic and trained care to the same extent. There is no work which puts greater demands on the moral qualities of a young nurse than the nursing of the aged and the imbecile, the guiding of the old folk as they go down into the Valley of the Shadow. There is no more beautiful and Christian scene than the sweet patient attendance of the infirmary nurse on her ward full of frail and fretful old dames. It is said that children cannot thrive without love; the infirmary nurse knows that often the old cannot die in peace without affection. How many of these aged patients turn to the nurse for the farewell kiss? And how often are the last words from the stiffening lips the only reward possible—"God bless you, Nurse!"

One story from the other side of the picture is sufficient; in one of the metropolitan unions it is customary if the women get their gowns wet and draggled when allowed out for the day, to send them to bed for three days as a punishment. It is very bad physically for old women to be kept in bed for three days when they are quite well: it is very bad morally to permit the officials to thus treat those in their charge. We commend this case to the inquiry of Mrs. Despard, Mrs. Furley Smith, and other women guardians. The whole tone of most workhouses is disciplinary and harsh; it has been universally admitted that it is unfit for children, and now the pauper child is boarded out or sent to scattered homes. The Kingston Guardians have also discovered that the real "work"house is equally unfit for the aged and the imbecile, and if they can carry their scheme into operation, no doubt other unions will follow in their footsteps. For the married couples cottages are supplied in many cases, but the bulk of the old men and women who end their days on the rates are widows or widowers, or spinsters, or deserted. Every workhouse has hundreds of these old people who are past work, except the light employment perhaps supplied by the Brabazon scheme. They sit round the rooms on forms put against the wall, all full of silent despair, or on fine days they totter out into the "yard," and sit or stand about round the little plot of grass. The unnatural seggregation in age groups makes it all very dreary: there are no grand-children to watch tumbling about on the grass, there is no married daughter to make a becoming cap for granny and give her something to think about; it is all a mechanical routine and a waiting for death. The officials are doubtless competent and see that all is clean, and there is a sufficiency of suitable food, but they have not got the nursing ideal before them, they have not got that sympathy which every good nurse is bound to develop, and they have not got the trained observation necessary for those in charge of the old. A nurse knows that it is not well to disturb the habits of the old; that they get used to certain things that would be injurious to the young. Dr. Bishop writes "You should not dictate to them as to the hours of rising nor retiring, nor too much as to what they should eat. A very old person gets into a groove and the nurse must not disturb it too much." The signs of senility, the rapid attacks of pneumonia, all these a trained nurse knows a little about, though we could wish there was one good book on the nursing of the aged to counterbalance the hundreds on the nursing of children. But certainly it is an advance that one Board of Guardians has recognised the right of trained attendance for the imbecile and the old.

There is a point, too, in this matter which is important to nurses and the public. Experience shows that the most popular nurses supplied by the corporations to the public are those who have been trained in Poor-law infirmaries. One reason for this is indicated in a leading article we publish elsewhere; but the main cause seems to be that hospital-trained nurses often lack the common sense and kindliness which a Poor-law infirmary nurse gains as part of her training.

### Lectures Upon the Nursing of Mental Diseases.

By Robert Jones, M.D.Lond., B.S., F.R.C.S.Eng., M.R.C.P.Lond., Resident Physician and Superintendent of the London County Asylum, Claybury.

### LECTURE XX.

In a previous lecture it was stated that the insane are subject to the same bodily diseases as the sane. Owing to the mental affection, however, these are by no means easy to diagnose, as little or no assistance can usually be given by the patient whereby the medical man and the nurse are enabled to appreciate the nature of the disease. Pain, which as a subjective symptom is a valuable index, may be altogether absent, or it may be misleading, and the only symptoms are those objective ones which are discovered by direct physical examination of the organ affected. It is important to differentiate between symptoms and signs; the former relate to changes of function in an organ, such as palpitation, shortness of breath, difficulty of moving, and they may or may not indicate disease in the particular organ affected. Thus, pain in the knee may point to disease in that joint or in the hip, and pain in the chest may indicate commencing pneumonia, pleurisy, heart-disease, or even a fractured rib; but when *crepitus* is obtained, by placing the hand, *i e.* by palpation, over the seat of pain we have a definite physical *sign* of fractured bone. Signs are unmistakable evidence, symptoms possible evidence. Signs of chest trouble may be obtained by auscultation, percussion and other methods of physical examination. Fulness of the abdomen in a woman of a certain age may indicate or may be a symptom of pregnancy, but the sound of the fœtal heart is a *sign.* Pain, therefore, as a symptom of disease, so much relied upon in the sane and which may be sharp or acute, a twinge, or a pang, heavy or weighty, full or tensive, shooting or lacinating, tearing or burning, is often entirely absent in the insane owing to mental impairment and may, when present, and in consequence of delusions, be misinterpreted. Insane persons often obstinately resent questioning and refuse to submit to any kind of physical examination, hence it is important for the nurse to note and report any change in the conduct or manner of her patient, so that early and proper treatment may be applied.

In this lecture we propose to deal with symptoms of disease as occurring within the chest, viz. of the respiratory and the circulatory systems, and only so far as concerns the insane, for the nurse is already considered competent to deal with ordinary sick nursing.

Pain as we have seen may be absent, and cough also, which is a reflex act, may never be present even throughout the whole course of ordinary *phthisis pulmonalis* (consumption) the expectoration not being coughed up but quietly swallowed, so that the first sign of phthisis may be diarrhœa, caused by tubercular affection of the intestine. The appetite may be undiminished, and the loss of weight be almost inappreciable. There are two symptoms, however, indeed they might be called signs, for they are definitely indicative of this form of lung affection, viz. (*a*) feverishness at night or evening temperature, and (*b*) dyspnœa or difficulty in breathing.

Whenever an insane person appears to be out of health, although pain and other symptoms are absent, the temperature should be taken night and morning. The value of the clinical thermometer cannot be over-estimated in the appreciation of disease accompanied with mental affection. It is the first, most reliable, and the best means of obtaining information. If a cough be present, and it is coarse and croaking, or attended with a whistling or crowing sound, then laryngeal disease or obstruction may be indicated, and I have more than once seen this as the only sign of early malignant disease of the larynx. If the cough be attended with wheezing sounds or rattling and difficult breathing there may be bronchitis. If the cough be short and sharp pleurisy or pneumonia may be indicated, whereas that of whooping cough is unmistakable. If dyspnœa or difficulty of breathing be present and the respirations are shallow and more frequent than 25 to 30 in a minute pneumonia may be present; if prolonged, difficult, and wheezing, then bronchitis, asthma, or heart disease may be indicated. A cessation of respiration for a few seconds, then a sudden recommencing—at first rapid and shallow, then slower and deeper and again stopping, a condition called Cheyne-Stokes respiration, is an indication of serious brain affection; a state of breathing which may occur before death from heart or kidney diseases. The commoner varieties of inflammatory affections attacking the air-passages and lungs include ordinary catarrh or colds, laryngitis, *i e.* inflammation in the larynx; bronchitis, when the bronchi are affected; pneumonia, when the lungs are affected; pleurisy, when the pleura or covering of the lungs is affected and pleurisy with effusion, when there is a collection of fluid or empyema when pus collects in the chest; asthma, when there is a distressing difficulty of breathing; and consumption or pulmonary phthisis, when one or both lungs are affected by the *tubercle bacillus*—a condition which requires special attention, more particularly when a number of persons are collected together as is necessarily the case in public institutions.

*Catarrh or cold* is probably the most common affection of the respiratory tract and may be the precursor of laryngitis and bronchitis. It is a congestion of the whole of the mucous membrane of the nose and throat, with plentiful secretion of clear glairy mucus, which later becomes green and thick. It commences with headache, passes through a stage of active secretion necessitating the use of many handkerchiefs and may end, if neglected, in bronchitis.

*Laryngitis* is a cold, primarily in the voice organ or voice-box, as it is usually called. The voice becomes harsh and husky; there may be a dry cough, with much discomfort and difficulty of breathing, which during the night may suddenly, in adults as well as in children, become dangerous.

*Bronchitis* may follow upon either of the above. It is an inflammation of the membrane lining the whole of the bronchial tubes, and is the prevalent complaint of elderly persons in cold weather. It is often the cause of the chronic winter cough and the difficulty of breathing from which many aged persons suffer. It may appear in an acute form as the result of a cold, with a high temperature reaching to 103° or 105°, with cough, expectoration, marked difficulty of breathing with wheezing, and a feeling of great tightness across the chest. The expectoration passes through the usual stages, being at first glairy, or watery and frothy; then, as the mucus changes into pus, the expectoration becomes greenish or greenish yellow and sticky.

The shortness of breath and the difficulty of breathing may become very marked, as the disease spreads to the smallest divisions of the bronchial tubes, finally involving the little air cells of the lungs and ending in fatal broncho-pneumonia.

*Asthma* may be associated with chronic bronchitis. There is considerable difficulty of breathing, the inspiration is long and deep with much wheezing, and expiration is also long and difficult. The nurse should observe whether inspiration or expiration is the more difficult. The feeling of suffocation attending asthma is more distressing than dangerous. Asthma sometimes takes the place of an attack of insanity; at any rate, the mental symptoms have been observed to abate with its onset. Asthma may come on quite suddenly in the night, and may follow indigestion or the taking of some inadaptable food.

*Pneumonia* is an inflammation of the substance of the lung itself. It means a filling up of the air cells with inflammatory products, and is usually the result of a cold or chill. At times it seems contagious, and to occur as an epidemic. It is an acute disease, coming on alone, as it were, although it very often supervenes upon paralytic states, after accidents when persons have been long bed-ridden, in heart or kidney disease, or after rheumatic and other fevers.

In the case of the insane and in the form of broncho-pneumonia it is the most common cause of death among old people; it occurs also among those who require to be fed artificially, particularly if they lie much on their backs, and for this reason it has been called "food pneumonia." In the case of young people suffering from depression with refusal of food it may occur within a few days of feeding with the cup, the milk trickling into the larynx and lungs causing the temperature to rise, for milk forms a favourable culture medium for germs, and such a state not infrequently ends in fatal broncho-pneumonia. Ordinary pneumonia, primarily attacking the lungs, commences after a day or two of undefined illness in a severe rigor, with a temperature of 104°. The breathing is rapid, there may be a short cough and pain in the side, the face is somewhat flushed, there may be some vesicles of herpes round the lips, and there may be expectoration, after a day or two, of sticky, reddish-brown sputum, which is described as "rusty," as it is the colour of old iron, from an admixture of blood with the mucus. The skin is dry, there may be some jaundice, especially if the pneumonia is on the right side, as the up and down movement of the diaphragm is impaired and the bile is no longer circulated through the liver into the gall-duct, but is absorbed into the general circulation: there is also a destruction of blood corpuscles in these attacks which helps to give the jaundiced hue. The tongue is brown, the teeth are covered with sordes, and incrustations appear on the lips. There is usually marked delirium, with tremulousness and picking at the bedclothes at night, and the temperature remains high for from seven to nine days. It may come down suddenly—which is called a *crisis*—and there is profuse perspiration, or it may fall gradually—which is a *lysis*—and the skin becomes moist, the symptoms gradually subside, and the patient recovers. In a large number of cases the course is less favourable, exhaustion increases, the sputum from "rusty" becomes "prune-juice" coloured caused by a greater admixture of blood, then it becomes greenish and fœtid, indicating gangrene; the patient's colour becomes more livid and dusky, the respirations more difficult and rapid, the restlessness increases, the pulse fails, and death ensues.

*Pleurisy* is an inflammation of the covering membrane of the lung and that lining the chest wall, which in health touch and rub over each other without pain. Pleurisy may occur from a cold or a chill; from injury, such as a fractured rib; or in the course of rheumatic and kidney diseases; but most frequently from tubercle in the course of, or as the commencement of, consumption of the lungs. Pleurisy is either "dry pleurisy," which is a slight roughening and inflammation of the pleura giving rise to a sharp "stabbing" pain in the side during respiration, which is easier when the patient lies on the affected side, thus preventing the movements which occur in respiration; or it is pleurisy with effusion, when fluid accumulates in the thorax, pressing upon the heart and lung, pushing both from their ordinary positions and compressing the lung so that it becomes airless. This fluid may amount to quarts in quantity, it may be clear watery fluid, or mixed with a slight amount of blood, or, it may be pus—in which case the pleurisy is described as an *empyema.* The symptoms are a raised temperature (102° to 103°), difficulty of breathing, pain, an occasional short cough without expectoration but with some lividity. There is a great danger from sudden death, which is the most common ending among the insane in fatal cases; to avoid movement or restlessness the patient is made comfortable by being propped up in bed or lying on one side, which is often the easiest position.

*Phthisis or Pulmonary Consumption.*—Of all the deaths that take place in asylums for the insane phthisis is responsible, either alone or in association with other causes, for nearly one-fourth. It is caused by the invasion of a germ, the *tubercle bacillus*, which attacks the substance of the lung causing a little local inflammation and the formation of a small nodule or tubercle the size of a boiled sago grain. Those nodules are either separate or collect together into large masses, eventually breaking down in the middle, causing cavities in the lungs and giving rise to the sputum in which the tubercle bacilli swarm. As pulmonary consumption is infectious and the actual cause is known, it becomes imperative and obligatory that no extension of this disease becomes possible in institutions where many people are collected together. The symptoms of phthisis, so well known in the sane, may be masked in the insane. There may be no cough, and it is often impossible for a medical man to make the thorough and complete examination which is possible in the case of sane persons. The patient will often neither breathe nor speak when told, but he will shout and resist all efforts at examination. The best tests in such a case are the body weight, the shortness of breath, and the morning and evening temperature, the so-called night sweats and diarrhœa. Spitting of blood, which is a common incident in the course of consumption, may be altogether absent in the insane or it may occur as a complication. The deposit of tubercle in other organs is also a complication occurring in cases of phthisis, and may take place in the intestines as stated, from swallowing the sputum; in the larynx, or in the membranes of the brain; the latter, however, is very rare except in young persons who have suffered from ear disease with discharges. Air in the pleural cavity may occur as a complication in cases of phthisis after sudden or severe exertion, and such a condition is accompanied by pain, difficulty of breathing, and collapse. It occurs owing to air passing through the diseased lung into the cavity of the pleura, thus causing the lung to collapse.

(*To be continued.*)

### Where to Go.

BAZAAR and sale of work in aid of Bolingbroke Hospital, Town Hall, Lavender Hill, Battersea, October 19th, 20th, and 21st. Opens at 3 P.M.

### Wants and Workers.

NURSE FOSTER, of All Hallows Mission House, 127 Union Street, Borough, S.E., wishes to enlist sympathy in the very sad case of a nurse, just over 40 years old, entirely without means, invalided with heart disease and nerve trouble resulting from it. Can anyone, she asks, suggest a home?

### Beyond the Seas: Nursing in the Australian Bush.

*Concluded from page 9.*

### An Incident.

One incident that happened at the camp will ever remain fresh in my memory. It was owing to the boy's brain trouble. At the time he was on the high road to recovery; but he was very weak both in body and mind. His little sister was still carried on to her couch in the garden, but he used to stagger there himself. I then left them in charge of each other while I looked after the mother, who was still helpless and in bed. One day,after fixing her up as usual, I hailed the children from the verandah. Imagine my feelings at finding that one of the birds had flown. The little girl was crying, and said that her brother had dared her to tell, but that he had gone to find fruit.

"Nonsense," I cried; "he can't get fruit about here, and he knows that it might kill him if he ate any."

"He knows," she said, "but he is mad for passion-fruit and figs, which are both ripe up at the old garden, and he means to get there somehow."

In a fright I ran down to the beach and looked up and down the glaring beach, across the torrid indigo water, and scanned the outlining dry bush in all directions. There was no sign of life anywhere. The earth seemed to have given itself up to scorch and simmer in the blazing sun, and cringe under the hot land breeze which was blowing. Running on up to the public-house, which was deserted save for one man, I asked if he had seen Billy. Yes, he had seen him in his boat putting the sail up.

"Nonsense, he was not strong enough to lift an oar, much less a sail, and how could he push off?"

"The dingy was floating nurse, and he got in off the jetty and managed to get the sail up. I watched him."

"He couldn't have done it alone, it is a physical impossibility; someone must have helped him."

"He did do it, though he was pretty dicky over it; once I made sure he'd go over, but the wind righted him."

"How could you let him?" I cried reproachfully.

"He told me you said he could go and fetch some fruit from the old garden up the river. I said I didn't think it fit. He only grinned all over his white face."

"There's a puffy wind," I remarked, turning and facing the broad estuary with my heart in my mouth. "And if he manages sail and helm in it, mad as he is, and reaches the fruit safely, that will kill him. Under the circumstances could you follow him?"

"Sorry that that is impossible. You see the boss left me in charge, and there is no one else about." A wild notion flashed through my brain that I should take charge of the bar while he went; but then I remembered that a ferry would be in, and chose the other alternative.

"Put me out a dinghy and a light pair of oars, though I can't row much, someone must go."

"The wind is dead against you, and the sun is terribly hot."

"So it is for the child," I commented, and ran up to the camp to prepare the mother. The servant was washing. The poor creature was crazed on some points, one being that when she was washing, nothing on earth would remove her from the tub until she had finished; not even the sun, when it came creeping round the corner of the shanty, and beat down on her poor sweltering body with its fierce rays. When I told her what had happened, she merely looked up, wiped the wet from her face, and said: "I'll come along when I've done." In despair of any help from that quarter, I took my little girl patient in beside her mother, and left her in charge, with a jug of milk beside her.

### An Inexperienced Boatwoman.

Off I set in the dinghy, feeling mighty clever till I got mixed up in a current, then the work began. I got broadside on, "caught a crab," and shipped a lot of water, soaking dress and feet. Then the wind got under my mushroom hat and played havoc with my hair, nearly tearing it out by the roots. Not daring to leave go of the oars with both hands to tie on the hat, I tore it off with one and shoved it under my knee. This made the boat drift several yards, and I had to set to and pull it up again. Though fairly righted then, I seemed to make no headway but it was no use giving in, so I pulled, and pulled, and pulled. My hair flew out on all sides, the sun baked my head, my back and knees and arms ached fearfully, yet I seemed to make no headway. Then I determined not to look over my shoulder any more, not to remember that I was I, rather that I was some struggling wild spirit, battling with a storm, trying to reach a small white face gleaming up at me from every white-crested wave I saw with the corner of my eye, as I leant back for the next stroke. Though I was in dire discomfort, I forgot the discomfort in trying to attain what my whole self was centred on gaining, and now my energy was so taken up with trying to reach the headland before me that I was surprised and overjoyed when at last I bumped into it, and there right beside me in the deep water was a sail. Hailing the occupant, I pushed off for it, but alas! it proved to be only one of the camp soldiers.

"Seen a little boy in a boat?" I cried.

"There's a boat round the headland."

"Near the old garden?"

"Yes."

"Was there a boy in it?"

"Couldn't swear. Ta-ta." He let his sail fill, and was off with the wind down stream. I vaguely wondered what he thought of "she" out in a boat—hair streaming, clothes wet.

### Capture of the Runaway.

At last I got round the headland, and there in a calm bay lay our boat with sail full rigged, and the boy with a defiant look on his face, sitting on the side, dangling his bare legs in the water. He smiled sardonically, when I drew alongside.

"What have you come for?"

"To join your picnic. Any fruit?"

"Not yet, I'm having a rest." I noticed his green pinched face. "I called to the old man who lives in the shanty, but he must be out pruning. You go and find him Nurse, I do want some figs."

"I want to rest, too, 'twas hard pulling against tide."

It was beautiful sitting there in the shade, with the green garden above us, and the greeny blue water at our feet. Some silvery fish glided through it, and a shag dived down near to catch them, while parrots chattered in the trees beside us. I waded round to the end of the boats to tie the two together.

"I won't have that old dinghy tied to mine," shouted the boy.

"It won't jar, the wind carries her out. Show me how to tie a proper knot."

He was proud of his seamanship and did so, then I sat watching his roving, hard eyes with the strange look in them and wondering if we should save his reason. Presently he looked up and sniffed. He was a grand sailor.

"The wind's changed," he chuckled. "We'll have a fine sail home."

"Lovely," I breathed. "Let's off now, while it fills the sails."

"Oh, you silly, you; 'tisn't filling the sail, it's dead against us where we are lying."

"Won't you show me, out there, how to sail with the wind behind us? I know I'm silly about a boat."

"You'll have to push off, then; we've run up on a sand-bank."

Obediently I slipped into the clear, shining water. It was delicious, but I had another piece of hard work before me, for push and push how I would I could not loosen the boat from the sand with the wind driving in on the sail. I was then ordered to get into the boat, furl the sail, and lift the mast down. This nearly finished me up, for I shinned the boy painfully and made him howl and swear, and I bumped my own head. However, we succeeded in getting off and putting the mast up again. How the boy had done it in the first instance will always remain a marvel. He then took the sail rope, turning it round his arm so that it was in one hand and the tiller in the other, while I sat on the seat near watching him anxiously, and the dinghy danced behind. The little fellow thoroughly understood a boat, but he was hopelessly weak. His face went from green to purple, and back again to green, round his eyes came dark rings, and he stooped forward, hanging on to the rope and let the tiller slide as if he were going to faint. If the wind had not been behind us, we would certainly have capsized, for I knew nothing about sailing.

### A Sick Man at the Helm.

These are the only remarks that passed between us during our perilous voyage back:—

"Let me take the sail down now, and row. You're tired."

"If you try to, I shall jump overboard and leave you to it."

. . . . . . . . .

"Look out! You're running straight for that spit-post. Ah! Clever boy; however did you clear it?"

"Shut up, you silly! Don't you know you oughtn't to speak to the man at the helm?"

. . . . . . . . .

"Oh, dear! . . . I thought we were over that time."

"Shut up! I'm going to jib. Duck!"

"Do take care, Billy! We're in that awful deep water where the currents are."

"Be quiet, can't you?"

. . . . . . . . .

"Do run up on the sand bank, and let me take down the sail before we go under the bridge. You know how hard it is to steer through the piles."

"Shut up! . . . Shiver me timbers! She got a nasty jar there though."

. . . . . . . . .

"There's the ferry! Oh! Billy, take care! You're going straight for it!"

"Shut up. I've told you to shut up before!" he screamed. "It's all your fault we're going into the ferry. I can't help it."

Like a shot I untwisted the rope from the child's arm, and, pushing him down in the boat, seized the tiller. All the time I had been working the thing out, and saw how it went. In a minute the sail jibbed, and we ran safely up on a bank close to the camp. The bar-man came to meet us, and took the boat in charge while I carried the boy to the camp. He was exhausted, and slept for hours afterwards. When he woke up he was quite sane, and declared he did not remember anything that had happened since his illness. Anyway, he was splendidly well after this, and I never had any more bother with him; he would follow me about like a dog, and do anything I told him.

### Central Midwives Board.

A meeting of the Central Midwives Board was held at the Board Room on Thursday, September 29.

In the absence of Dr. Champneys, Mr. Parker Young was in the chair. The other members present were Miss R. Paget, Miss Oldham, Dr. Sinclair, and Miss Wilson.

The correspondence included a letter from the secretary of the London Obstetrical Society with regard to the case of a certified midwife whose certificate the society had resolved should be forfeited. The Board agreed to call for some further evidence before taking any action. A Cardiff doctor wrote complaining of the conduct of a certified midwife; the consideration of this case was postponed. Some discussion occurred in reference to a letter from the medical superintendent of the Brentford Union, asking for advice on the position of new lying-in wards. Dr. Sinclair considered that such questions were entirely outside the scope of the Board, and wished a reply to that effect to be sent, but this view was not supported, the other members being of opinion that the Board should not snub those who asked advice, but give it where possible. On the motion of Miss Wilson, seconded by Miss Oldham, it was decided that a letter, recommending the medical superintendent to inspect the Kensington Infirmary lying-in wards, should be sent, Dr. Sinclair protesting that the Board was making itself ridiculous. Another letter led to a somewhat similar discussion. The Town Clerk of Poplar wrote urging the Board to promote legislation to empower local supervising authorities to grant compensation to midwives suspended from practice under Section VIII. (3) of the Act. Dr. Sinclair again objected to the Board taking any part in matters beyond its legally defined duty of administering an Act of Parliament, and desired to dissociate himself from these irrelevancies. Mr. Parker Young said he thought that it was not outside the functions of the Board, but good sense to note anything wrong in connection with the working of the Act, and promote amendment by legislation if need be. At the instance of Miss Wilson, seconded by Miss Paget, it was resolved that the secretary should make a note of the Poplar letter for future reference.

The applications of 1,534 midwives for certificates were approved.

An application from the Newcastle-on-Tyne Lying-in Hospital, for approval of certificate under Section 2 was granted.

The following institutions were approved for the training of midwives under Section C of the rules:—The Rotunda Hospital, Dublin; the Clapham Maternity Hospital; and the Aberdeen Maternity Hospital. In several instances where newly-formed institutions asked for recognition it was resolved to send letters expressing the hope that favourable consideration might be given to their applications when in full working order. Miss Wilson and Miss Paget thought that it was most desirable to give every encouragement to the efforts being made to establish training centres; Miss Paget especially, as the representative of the Queen Victoria Jubilee Institute, speaking of the maternity branch in course of formation at Cardiff. Dr. Sinclair was again in opposition. He considered that there were no representatives on the Board, and that such information was mere gossip.

The Board next dealt with applications for recognition as "teachers" under Rule C.I. (3). Dr. Sinclalr hoped that certain principles would be definitely followed in approving these applications. For example, he said that it was undesirable in any case for medical officers of health to be approved as teachers, with which the Board agreed. A

number of names were approved, others postponed or rejected. The applications for approval as certified midwife for the purpose of signing forms III. and IV. under Rule C.I. (2) were postponed to allow of notice to be given by Miss Paget of a resolution limiting such approval to one year.

Mr. Ward Cousins not being present, a resolution in his name was also postponed.

### Everybody's Opinion.

[Correspondence on all subjects is invited, but we cannot in any way be responsible for the opinions expressed by our correspondents. No communication can be entertained if the name and address of the correspondent are not given as a guarantee of good faith, but not necessarily for publication. All correspondents should write on one side of the paper only.]

### STATE REGISTRATION.

"Anxious" writes:—Seeing Mr. Sydney Holland's offer to supply any nurse with a copy of his evidence against registration, and a list of signatures against the Bills, I applied for a copy. I was in favour of registration, but wished to hear both sides of the argument. May I say that I find the arguments most convincing, and I advise nurses who are studying the question to obtain a copy from the London Hospital for themselves? The experience of those who have signed against the Bills is of value and not to be lightly set aside. I trust that you will find space for this, as it is a most important question for private nurses. As they would be principally affected by legislation it is for their own interest to hear both sides.

### DRESSINGS AND ECZEMA.

"A District Nurse" writes:—I should be glad to know the general custom as to supplying dressings for surgical district cases. Is it usual for the doctor to supply club cases and patients from the works who pay so much a week or per quarter for the doctor's attendance, or is the district chargeable? The doctor supplies dressings to cases able to attend at the surgery. Should not some of the dressings, at any rate, be provided for the patient confined to bed and attended by the district nurse, or is it usual to expect club cases &c. to supply own dressings? If so, then, of course, if patient is too poor to buy dressings the district supplies. Has any nurse found a cure for eczema—not of the head, but long-standing eczema of arms and legs? Ung. zinc. and hyd. nit. dil. I have found useless beyond a certain stage.

### FULL INDOOR AND OUTDOOR UNIFORM.

"A Nurse's Friend" writes: Will you or one of your readers kindly inform me what is meant by "full indoor and outdoor uniform" for a nurse? Some friends of mine, after completing their training, joined a nursing institution, and were informed that in addition to the agreed salary "full indoor and outdoor uniform" would be provided. All they had given them was the material for dresses and cloaks and one bonnet. The making, trimming, buttons, etc. have to be paid for by the nurses, who also have to find their own caps, aprons, collars, and cuffs. I consider that this is most unjust and an absolute breach of agreement; but what newly joined nurse would care to make a fuss and risk getting in the "black books" of the principal at the commencement of her engagement? Would the principal, however, consider a nurse in "full uniform" if she went to a case with a few yards of dress material tied round her with, say, a piece of string? And yet that is all that is provided. I hope that the publication of this letter may be the means of stopping such a serious injustice.

### Appointments.

[No charge is made for announcements under this head, and we are always glad to receive, and publish, appointments. The information, to insure accuracy, should be sent from the nurses themselves, and we cannot undertake to correct official announcements which may happen to be inaccurate. It is essential that in all cases the school of training should be given.]

Bermondsey Infirmary.—Miss Mabel Herbert and Miss Charlotte Ann Allen have been appointed charge nurses. Miss Herbert was trained at Shoreditch Infirmary, and since her training has been charge nurse at Portsmouth Infirmary. Miss Allen was trained at Swansea General Hospital, and has since been on the private staff. She has also been staff nurse at the Cottage Hospital, Bromley, Kent, and private nurse at Bath.

Dumfries and Galloway Royal Infirmary.—Miss Annie Proctor has been appointed sister. She was trained at Ancoats Hospital, Manchester, where she has since acted as sister.

Fever Hospital, Mill Lane, Liscard.—Miss Blanche Sloane-Ashman has been appointed sister. She was trained at Crumpsall Infirmary, Manchester. She has since done private nursing, and has lately been assistant nurse at the City Fever Hospital, Grafton Street, Liverpool.

Johnstone Combination Hospital for Infectious Diseases.—Miss Isobel M. Rylands has been appointed nurse. She was trained at Crumpsall Infirmary, Manchester, and has since been attached to the Dublin Trained Nurses' Institute; St. Cuthbert's Hospital, Edinburgh; the Eastern Fever Hospital, London; and the Hillhead Nurses' Institute, Glasgow.

Nover's Hill, Hospital, Bristol—Miss Eugenie B. H. Scott has been appointed night superintendent. She was trained at the Western Infirmary, Glasgow.

Stirling District Asylum, Larbert, N.B.—Miss Caroline Fielding has been appointed assistant matron. She was trained at Bradford Union Infirmary, where she has since been theatre nurse and ward sister. She has also been ward sister at Stobhill Hospital, Springburn, Glasgow. She holds the L.O.S. certificate.

### TRAVEL NOTES AND QUERIES.

By our Travel Correspondent.

Italian Lakes in November (Maud).—I should advise you to spend one fortnight at Cadenabbia, on Lake Como, and one fortnight at Baveno, on Lake Maggiore: these two places are on the whole the best centres. At Cadenabbia Hôtel Pension Cadenabbia is very reasonable: terms from 7 francs per day. Hôtel Britannia, is much the same, but a trifle dearer. At Baveno Hôtel Pension Suisse cheap. Offer 4½ francs and see if it is accepted. If that house be full, go to Hôtel Beaurivage. The cheapest route is *via* Newhaven and Dieppe, Bâle and the St. Gothard: second-class return £6 10s. 6d. Be sure to take plenty of warm clothes. It can be very cold on the lakes in November, and you will miss the comfort of fires. Take also a spirit kettle, materials for making tea, and do not omit rubber hot bottles. Let me hear how you get on.

Bay of St. Malo (C. H. B.).—Many thanks for information concerning Mrs. D. It is always acceptable to have personal testimony to the excellence of a house. I know this one well and often recommend it. It would not, however, suit the correspondent to whom I replied when you noticed the answer.

Cheapest Route to Clarens (Francesca).—That *via* Newhaven, Dieppe, and Pontarlier is the cheapest: second-class return £4 13s. 6d. *Via* Dover and Calais it would be £6 1s. Write to Messrs. Cook and Sons, Ludgate Circus, for your ticket, and mention that you wish to break the journey at Paris. You can do so and continue your journey with the same ticket.

### Echoes from the Outside World.

### Movements of Royalty.

The King returns to London from Balmoral this week. On Monday his Majesty and party had an excellent day's trout-fishing on Loch Muick. During the day the two sons of the Prince of Wales visited the King in order to take leave of him before their departure for London. The Queen is still in Denmark, but returns to England next week. The Princess of Wales and her children arrived at Marlborough House on Wednesday evening.

### The War in the Far East.

The first Japanese train has arrived at Liau-yang, and the completion of the railway entirely removes the problem of the transport of supplies for the Japanese forces in the field. There is no news from the front in Manchuria, except a short report from General Sakharoff to the effect that the Japanese advanced posts are increasing in strength, and a statement that in the higher military circles in St. Petersburg a serious Japanese offensive movement against Mukden is expected early next week. Their forces are stated to amount to about 144,000 infantry, over 6,000 cavalry, and 600 guns. General Kuropatkin has addressed to the Russian Ministry of War a letter bearing emphatic testimony to the good service rendered by the commissariat. The Japanese Premier declared at Tokyo on Monday evening that the war would continue for a long time.

### Death of Sir William Harcourt.

The death of Sir William Harcourt occurred, suddenly, at his residence, Nuneham Park, Oxfordshire, early on Saturday morning. He retired to rest on Friday night, without any complaint of indisposition, and passed away peacefully in his sleep. The event was so unexpected that only Lady Harcourt was with him at the time, neither Mr. L. V. Harcourt, M.P., nor Mr. Robert Harcourt, his sons, being able to arrive at Nuneham before he died. Sir William Harcourt was born on October 14th, 1827, and was therefore within a fortnight of his 77th birthday. His grandfather was at one time Archbishop of York. He was 41 when he entered the House of Commons and 53 when he became Home Secretary in Mr. Gladstone's Government. He was subsequently Chancellor of the Exchequer in two Administrations; and when Mr. Gladstone retired from party life he became the acknowledged leader of the Liberal Party, a position from which he formally withdrew in 1898. At the time of the Coronation he refused a peerage. Among the telegrams of condolence received by Lady Harcourt was one from his Majesty the King, who at a quarter to three o'clock on Saturday afternoon telegraphed from Balmoral: "Allow me to express my deepest sympathy in the sad loss you have sustained. I have lost an old and valued friend in your husband." His Majesty has since sent an autograph letter of condolence to Lady Harcourt. The death of the distinguished statesman has provoked appreciative tributes from organs of all shades of politics.

### Lord Roberts in South Africa.

On Friday Lord Roberts, who, in company with Lady Roberts, is visiting in South Africa, inspected, by the aid of a plan, the battlefields and positions at Magersfontein. The proprietor of the farm at Magersfontein at the time of the battle pointed out the dispositions of the troops, and Lord Roberts ascended the kopje and saw the memorial to the Highlanders who fell on the occasion. He expressed appreciation of the work done by the Women's Guild in tending the graves. On the same day, in the Town Hall at Kimberley, Lord Roberts was presented with two rough diamonds, the people's birthday gift. Fifty children who were born during the siege of Kimberley drew lots for the honour of making the presentation. The Mayor's daughter, who was also born during the siege, presented an album of siege views to Lord Roberts, who, after he had responded, was photographed on the steps of the Town Hall in the midst of the siege children.

### Lady Curzon's Illness.

The illness of Lady Curzon took a favourable turn last Saturday, and it was announced on Sunday morning that in future only morning bulletins would be issued. The patient was so much better on Monday that her mother and sister, on their arrival from the United States, were able to see her. Sir Thomas Barlow and Mr. Watson Cheyne left Walmer Castle the same day, and Dr. Champneys has since been in attendance. Tuesday's report was less satisfactory, but on Wednesday morning it was stated that her condition was somewhat better.

### Resignation of the Governor-General of Queensland.

Sir Herbert Chermside, who was appointed Governor-General of Queensland in 1902, has resigned his appointment. His term of office does not expire until 1906, but he now retires for domestic reasons. Lady Chermside, who has been in England for some time, found it almost impossible to reside in Brisbane owing to the condition of her health. The climate, it is stated, is not sufficiently bracing for her, and she was soon compelled to absent herself from social functions at Government House.

### The New Lord Mayor of London.

The election of the new Lord Mayor of London took place on Michaelmas Day, and the choice of the Court of Aldermen fell upon Mr. Alderman John Pound. The Lord Mayor-elect was born in 1829 at 81 Leadenhall Street, the premises in which he still carries on business. He was educated at Christ's Hospital, and subsequently joined his father's firm. In 1869 he entered the Corporation as a Common Councilman, and he has been an Alderman since 1892. In 1856 he married Miss Harriet Lulham, and there is a family of two sons and three daughters. For the thirtieth year in succession Sir William Soulsby will serve as private secretary to the Lord Mayor.

### Railway Accident in Wales.

A terrible railway disaster occurred on Monday near Llanelly to the morning Great Western express train from Milford to London. As yet no reason can be assigned for the accident. A sudden jerk was felt, as though the locomotive was running over something rough, and then it went on for a distance of 100 to 150 yards when it pulled up. As soon as the uninjured passengers got out of their carriages it was seen that the first engine had left the rails, killing both the engine driver and the fireman, the former being quite decapitated. The second engine had remained on the line, but the two next carriages had gone right over a low embankment. They were completely smashed and had carried the line with them, the metals being twisted about like thin wire. It was impossible, at first, to summon help, but fortunately a doctor was among the passengers and he rendered splendid assistance. His supply of bandages was so limited that he ultimately tore up his coat to provide what was necessary. A soldier whose eye was hurt, his hand smashed, in addition to internal injuries, refused to be seen to till those in worse plight than himself had been relieved and even managed with his two companions to assist, remarking that they "had had to put up with worse things in the South African War." Three persons were killed on the spot and between 30 and 40 injured. An inquest on the bodies of the dead was opened on Tuesday, and adjourned to October 26th.

### Notes and Queries.

**FOR REGULATIONS SEE PAGE 12.**

*Private Nursing in New York.*

(7) I should be much obliged if you could give me any information as regards private nursing in New York, U.S.A.—*J. M. K.*

We cannot give you any special information about private nursing in New York, but that city is already well provided with American nurses, and no English nurse should go out there on speculation.

*Books and Fevers.*

(8) Can you tell me of a good book on fevers in the tropics, and also who is the best doctor on West Coast fever?—*Sister Elaine.*

"The Nurse in Hot Climates," by Dr. E. Henderson. Published by the Scientific Press. We cannot undertake to recommend consultants.

*Hospital Training.*

(9) Will you kindly tell me if a two years' certificate for general training and one for a year's training in fever would under the proposed new registration regulations equal a three years' certificate?—*W. M.*

It is impossible to say what might happen under future legislation which at present is only a matter of talk. But it is unlikely that the three years' certificate for general training will be interfered with.

*Salary.*

(10) Will you kindly tell me what would be a reasonable salary for a midwife holding a situation as District Maternity Nurse with night and day work? She would provide her own board and rooms, attend to about 240 cases in the year, visit and superintend the nursing, and act as midwife in about 100 cases, of which a large proportion would be abnormal.—*May.*

Write to the Secretary of the Association for the Training and Supply of Midwives, Dacre House, New Tothill Street, Westminster, S.W.

*Lecturer.*

(11) Will you kindly tell me where a trained nurse can gain a certificate as to her ability to lecture on hygiene, infant feeding, and kindred subjects? Would the certificate of the Sanitary Institute be of use?—*M. G. F.*

(1) See the Nursing Outlook in The Hospital for Sept. 24. (2) Yes.

*Homes.*

(12) Will you kindly tell me of a home where a woman of 76 (able to wait upon herself) could be received? She has no means whatever, but a daughter, who has to earn her own livelihood, would most willingly pay a small sum weekly.—*District Nurse.*

The Homes for the Aged Poor might receive the case. Write to the Secretary, Miss E. G. Harrison, 86 Penge Road, Anerley, S.E. Payment about 4s. a week. Or St. Peter's Home and Sisterhood, Mortimer Road, Kilburn, N.W., which only receives women and children, the boys being under seven.

The Vicar of my parish has asked me to try and find a home where a girl who is gradually becoming blind could be received. She also suffers from hysteria. She is 24, an orphan, but has an uncle who would probably contribute to her support.—*R. E. G.*

Provided that she suffers from no general organic nervous disease the Secretary, the British and Foreign Blind Association, 206 Great Portland Street, W., might be able to advise you. Enclose stamp for reply.

*Naval Nursing Service.*

(13) Can you kindly tell me to whom I should apply for information regarding the service of naval sisters?—*L. K.*

Write to the Director-General, Queen Alexandra's Royal Naval Nursing Service, 18 Victoria Street, S.W.

**Handbooks for Nurses.**

"A Handbook for Nurses." By Dr. J. K. Watson. 5s. net; 5s. 4d. post free.

"A Complete Handbook of Midwifery." By Dr. J. K. Watson. 6s. net; 6s. 4d. post free.

"The Nurses' Pronouncing Dictionary." 2s. post free.

"Elementary Anatomy and Surgery for Nurses." Price 2s. 6d.

How to Become a Nurse: "The Nursing Profession." 2s. 4d. post free.

The above may be obtained of all booksellers or of The Scientific Press, Limited, 28 & 29 Southampton Street, Strand, London, W.C.

### For Reading to the Sick.

"FAITH, HOPE, AND LOVE."

Have Faith: where'er thy barque is driven—
The calm's disport, the tempest's mirth,
Know this—God rules the host of heaven,
The inhabitants of earth.

Have hope; though clouds environ now,
And gladness hides her face in scorn;
Put thou the shadow from thy brow—
No night but hath its morn.

Have love: not love alone for one,
But man as man thy brother call,
And scatter like the circling sun
Thy charities on all.

Thus grave these lessons on thy soul—
Faith, hope, and love—and thou shalt find
Strength when life's surges rudest roll,
Light when thou else wert blind.

*Schiller.*

Depression and low spirits are a state which we all of us from time to time have to face! There will be, for all of us, times when, so to speak, the sun does not shine and things look dark and dreary round about us, and we feel as if we can only say *De profundis*, "Out of the deep have I called unto Thee, O Lord," or say with another Psalmist, "My soul truly waiteth in stillness upon God." Times when a nameless, inexplicable dread settles down upon us, and we know not how to face life or to go on! The sense of our deplorable weakness overwhelms us; the high aims and aspirations contrasted with the little that we actually accomplish! The question, at such times as these, is whether we are holding on to God in faith and hope an[illegible]ove.

How many—not to speak of m[illegible] serious sicknesses—how many have to suffer from some weakness of body which first seems to prevent them doing what others are able to do. "They are not strong—they are invalids—they are like maimed animals who, not having the full use of their bodily faculties, cannot have the full enjoyment of life." Life for them is rather dragged out than lived out buoyantly and strongly.

Into all this human experience of the perplexity of life, of the sense of chaos and incompleteness, our Lord entered—entered in fullest and completest sympathy. It is as if He said, "I know what this discipline means to you men and women, how small a part of God's plans you see: I have gone through it! I have allowed My human soul to be bereft of the consciousness of the Presence of the Father, and it was in fullest sympathy and love that I allowed Myself to cry out, 'My God, My God, why hast Thou forsaken Me?'"—*Randolph.*

If souls are right-minded and in their trials seek God more earnestly, if their pains drive them to His feet and make them feel their full dependence on Him, then, indeed, not an hour is lost; a great work is going on silently but surely; the heart is weaned from earth and fixed only on eternity. Whereas, when once a soul allows itself to be unstrung by the friction of trouble and departs from its true fount of consolation, once it tries to quench its thirst at fountains of its own making and seeking, then indeed much is lost that never can be recovered. "O my God, bid me cling to Thee! Give me patience under my sufferings and a happy issue out of all my afflictions."—*Anon.*

